# Predictors for adherent behavior in the COVID-19 pandemic: A cross-sectional telephone survey

**DOI:** 10.3389/fpubh.2022.894128

**Published:** 2022-10-20

**Authors:** Andrea Siebenhofer, Clemens Könczöl, Klaus Jeitler, Daniela Schmid, Phillip Elliott, Alexander Avian

**Affiliations:** ^1^Institute of General Practice and Evidence Based Health Services Research, Medical University Graz, Graz, Austria; ^2^Institute of General Practice, Johann Wolfgang Goethe University Frankfurt, Frankfurt, Germany; ^3^Institute of Psychology, University of Graz, Graz, Austria; ^4^Institute for Medical Informatics, Statistics and Documentation, Medical University Graz, Graz, Austria; ^5^Austrian Agency for Health and Food Safety Ltd. AGES, Vienna, Austria

**Keywords:** COVID-19, adherence, health belief model, social norms, self-efficacy, risk perception, perceived health risk, pandemic fatigue

## Abstract

**Background:**

During the COVID-19 pandemic, protective measures have been prescribed to prevent or slow down the spread of the SARS-CoV-2 virus and protect the population. Individuals follow these measures to varying degrees. We aimed to identify factors influencing the extent to which protective measures are adhered to.

**Methods:**

A cross-sectional survey (telephone interviews) was undertaken between April and June 2021 to identify factors influencing the degree to which individuals adhere to protective measures. A representative sample of 1,003 people (age >16 years) in two Austrian states (Carinthia, Vorarlberg) was interviewed. The questionnaire was based on the Health Belief Model, but also included potential response-modifying factors. Predictors for adherent behavior were identified using multiple regression analysis. All predictors were standardized so that regression coefficients (β) could be compared.

**Results:**

Overall median adherence was 0.75 (IQR: 0.5–1.0). Based on a regression model, the following variables were identified as significant in raising adherence: higher age (β = 0.43, 95%CI: 0.33–0.54), social standards of acceptable behavior (β = 0.33, 95%CI: 0.27–0.40), subjective/individual assessment of an increased personal health risk (β = 0.12, 95%CI: 0.05–0.18), self-efficacy (β = 0.06, 95%CI: 0.02–0.10), female gender (β = 0.05, 95%CI: 0.01–0.08), and low corona fatigue (behavioral fatigue: β = −0.11, 95%CI: −0.18 to −0.03). The model showed that such aspects as personal trust in institutions, perceived difficulties in adopting health-promoting measures, and individual assessments of the risk of infection, had no significant influence.

**Conclusions:**

This study reveals that several factors significantly influence adherence to measures aimed at controlling the COVID-19 pandemic. To enhance adherence, the government, media, and other relevant stakeholders should take the findings into consideration when formulating policy. By developing social standards and promoting self-efficacy, individuals can influence the behavior of others and contribute toward coping with the pandemic.

## Introduction

Since the beginning of the pandemic in December 2019, Coronavirus disease 2019 (COVID-19) has presented a significant challenge to health care systems around the world, with the numbers of hospitalizations due to COVID-19 diseases frequently surpassing system capabilities. In order to slow down transmission rates, almost every government in the world has developed a prevention strategy involving, for example, the use of face masks, hygiene guidelines, and social distancing (including stay-at-home orders), adherence to which was also recommended by the World Health Organization (1).

To develop and implement effective measures, it is important to obtain information on knowledge about COVID-19 in the broader population, and on peoples' attitudes and willingness to adhere to restrictions and recommendations (2). In addition to a recently published systematic review, meta-analyses involving a large number of quantitative studies published worldwide between January 1 and June 30, 2021, showed that at least 70% of questions about knowledge and what constitutes good attitudes and practice with regard to prevention-orientated behavior were answered correctly (3). However, people from low-income countries, men, younger people, and less educated persons generally had lower standards of practice. Another review published by Wake in 2020 also showed that the majority of the study population had a high level of knowledge, a good attitude, and high standards of practice. Moreover, besides variables such as marital status and media consumption, the study revealed the significant influence of age, gender, educational status, and income (4).

For management of the pandemic to be effective, it is important that epidemiological measures are adhered to. However, during the course of pandemics, willingness to comply with measures may change. A large cohort study in the UK involving the analysis of the patient data of more than 50,000 persons during two waves of the pandemic showed that most individuals complied with prevention behaviors (5). Data published by the Austrian Corona Panel during the first 10 weeks of the first wave in spring 2020 revealed that at least two-thirds of participants believed that measures introduced by the government were appropriate. But levels of agreement to all individual measures decreased steadily over the period (6). The COSMO-Spain Survey also showed that the level of adherence was considerable during three rounds of measurements from July to November 2020, and compliance with the mandatory use of facemasks reached ≥80% in all three periods (7). This is consistent with the results of the UK population study which showed that mask wearing was the most accepted measure (5).

The health belief model is widely used to develop a conceptual understanding of individual adherence to preventive activities (8–12). The basic assumptions of this model are that people are more likely to show certain health behaviors if they perceive a high risk of falling ill (perceived susceptibility), if the disease is perceived as serious (perceived severity), if those adopting the behavior see an advantage for themselves (perceived benefits), and if the obstacles to assuming this behavior are not too high (perceived barriers). Other important aspects of this model are a person's self-efficacy expectations and whether the person has been exposed to convincing arguments (cues to action) (13, 14). Lessons learned from previous pandemics such as swine-origin influenza (15), SARS (16), and EBOLA (17) also indicate that factors such as an individual's perceived risk, self-efficacy, and knowledge play an important role in adherence to preventive strategies.

These days, the health belief model is also used in SARS-CoV-2 research. Previous research on factors modifying adherence to protective measures to contain the COVID-19 pandemic show that an individual's perception of certain aspects of the health belief model and his or her preventive behaviors are influenced by social aspects, sociodemographic characteristics, and attitudes. Research shows that trust in science, government and administration, the media, and in the capabilities of the health system, has a significant impact on health behavior in connection with COVID-19 (18–21). Inconsistent results have been found for socio-demographic variables such as age, gender, education (22–27), and social norms (20, 28, 29). In one study published by Eichenberg et al. based on an online survey conducted in Austria, participants were categorized into four groups depending on their perceived susceptibility and their engagement in health-promoting behaviors (30). All four groups differed significantly with regard to almost all personality dimensions. Those who underestimated the COVID-19 pandemic and those for whom protective measures led to high emotional discomfort and stress showed significantly lower adherence to protective measures. In contrast, those with high levels of positive personality traits and that also considered governmental measures as appropriate, and those for whom the virus presented a danger and whose health depended on the effectiveness of the measures, were significantly more compliant. Data from Macao, China from a telephone interview study with 617 people in April 2020 (24) showed that the variables perceived benefit, exposure to a cue to action, perceived severity, and reward for use, were positively associated with a number of precautionary measures (wearing a face mask, proper handwashing, social distancing, avoiding touching one's face, flushing a toilet properly, and carrying a hand sanitizer). On the other hand, perceived barriers and social distancing were negatively associated with several protective measures. Most recently in December 2021, the Austrian Corona Panel published data collected consecutively over the first 12 months of the pandemic showing that people with lower health risk perceptions, less respect for social norms, and lower levels of trust in institutions were less likely to adopt preventive behaviors (31).

Another aspect that has frequently been examined in connection with the pandemic is corona fatigue (5, 32–34). The WHO defined pandemic psychological fatigue as a feeling of distress or frustration due to “sustained and unresolved adversity” (34) which is a feeling of tiredness of the pandemic and emotional exhaustion. According to a longitudinal telephone survey from January to December 2020 of over 30,000 persons (33), low public confidence in the government had a negative impact on precautionary behaviors and was associated with greater psychological fatigue. In contrast to other influencing aspects, corona fatigue changes over time.

All these papers aimed to examine potential mitigating factors to the introduction by governmental and stakeholder institutions of further recommendations to improve pandemic control. Based on the health belief model in a representative population in two states (Carinthia and Vorarlberg) in Austria, the aim of this study is to confirm known and identify new factors influencing adherence.

## Methods

Cross-sectional data from telephone interviews with 1,003 people living in Austria during the COVID-19 pandemic in spring 2021 were used for the analyses.

### Health belief model

**Figure 1:**
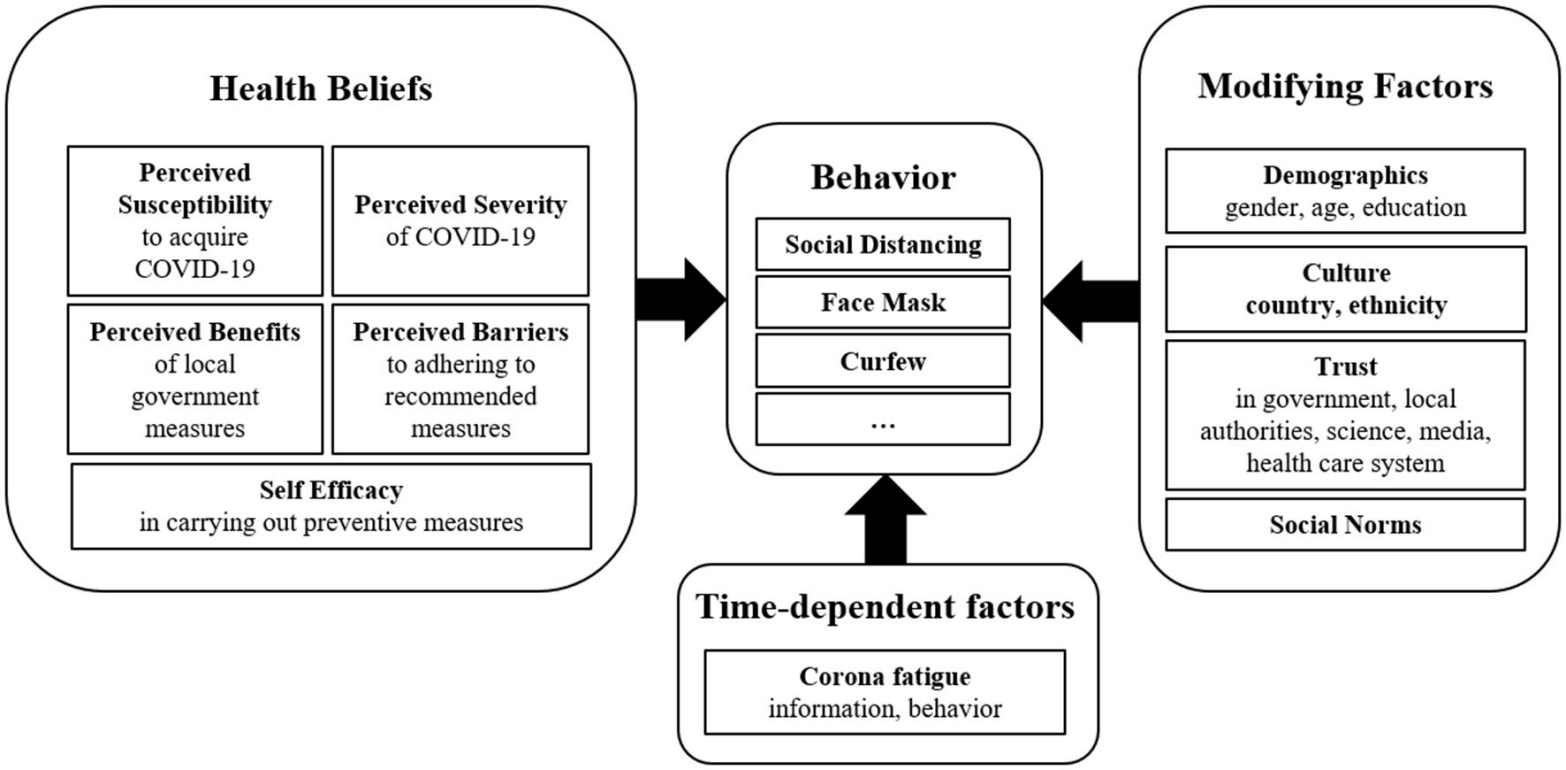
Model for explaining adherent behavior, based on the health belief model, modifying aspects, and time varying aspects.

We used the health belief model (HBM) adapted for use in COVID-19 research by Hsing et al. (35) and further expanded it by taking into consideration potential modifying aspects such as demographics, and time-dependent aspects such as corona fatigue. Figure 1 shows the key components of the HBM model used in this project.

### Questionnaire

The presented model (Figure 1) considers behavioral aspects and attitudes. These aspects also take the respondent's knowledge into consideration. The KAP-survey concept (knowledge, attitude, practice) was therefore used in the development of the questionnaire (2). To create an item pool for the COVI-Ad questionnaire, a literature review was carried out. Questionnaires that were based either on the health belief model or single aspects of it, and that had already been used during the COVID-19 pandemic and other pandemics or epidemics, were screened. New items were formulated for aspects that were not covered in these questionnaires. To make it easier to respond to the items during telephone interviews, the number of response categories was kept to a minimum. The resulting questionnaire was discussed within an expert group meeting (psychologist, medical doctors). After minor changes, eight telephone interviews were carried out in advance to assess the comprehensibility and feasibility of the questionnaire. The final questionnaire consisted of 68 items with a closed- and two items with an open-response format. A translated version of the German questionnaire can be found in the Supplementary material. Since the aim of the questionnaire is to map the relevant aspects of the adapted health belief model, the items were analyzed separately for each aspect. Explorative factor analysis (VARIMAX rotation) was carried out separately for all aspects apart from sociodemographic variables and single-item aspects. Internal consistency (Cronbach's alpha) was calculated for each resulting factor.

#### Behavior

Adherence to COVID-19 measures was assessed on the basis of six items (response format yes/no)—social distancing (refraining from meeting a large number of people), physical distancing (keeping distance to other people), respecting a curfew from dusk to dawn, wearing FFP2 masks, testing, and testing when symptoms are present. Participants were additionally asked if they had ever ignored any of the measures being assessed. As a result of factor analysis of these six items, four could be assigned to factor adherence. These measures were social distancing, physical distancing, respecting a curfew from dusk to dawn, and wearing FFP2-masks (Cronbach's α = 0.681).

#### Health beliefs

Five aspects of the adapted health belief model were measured using 18 items. To assess *perceived severity*, respondents were asked to compare COVID-19 to influenza (response format: harmless/comparable/more dangerous). Furthermore, the personal health risk and economic risk resulting from measures to combat the coronavirus were assessed on a Five-point Likert type response scale. No satisfactory result could be achieved in the factor analysis of perceived severity. All three perceived severity items were therefore analyzed separately. *Perceived Susceptibility* was assessed using a single item (response format: not at all/slightly/high). The aspect *Perceived barriers due to health-promoting measures* consisted of seven items (response format: yes/partly/no) and asked respondents whether they thought the measures were annoying, excessive, would be able to prevent the virus from spreading, had been scientifically proven to be effective, were constitutional or violated legal regulations, were feasible in reality, and whether they limited everyday activities. The first five items could be assigned to one factor (Cronbach's α = 0.792). The other two items were assigned to another factor (practicability of health-promoting measures), which, however, had too little internal consistency (α = 0.281) to be considered in the further analysis. *Incentives to engage in health-promoting measures* were assessed to ascertain the perceived benefits of health-promoting measures, whereby the respondents were first asked whether they considered the measures to make sense. For measures that were not considered to make sense, respondents were asked how likely it is that they would adhere to them (response format: quite likely, sometimes, quite unlikely) when adherence to the measures was officially checked, when high penalties existed for non-adherence, when someone they trusted could justify use of the measures, and when significant scientific evidence confirmed effectiveness. All these aspects were included in the resulting factor (Cronbach's α = 0.744). To measure *self-efficacy*, the respondents were first asked whether they considered the measures to make sense. For measures they considered to make sense, respondents were asked how likely it was that they would adhere to them when they were in the company of friends that were not (response format: quite likely, sometimes, quite unlikely).

#### Modifying factors

The following demographic variables were assessed: age (years), gender (female, male, other), living situation (living with children: yes/no, living alone: yes/no), employment status [retired, unemployed, self-employed, employed, short-time work, homemaker, parental leave/sabbatical/care leave, student (school, university, etc.)]. Educational levels were divided into five groups. EL1: Compulsory education including school leavers with no certificate of education, EL2: Apprenticeship, EL3: College for higher vocational education, EL4: Academic secondary school, EL5: University. The influence of culture was measured according to migration background (both parents born outside Austria).

For the **trust** aspect, respondents were asked whether they trusted information on corona that stemmed from politicians (prime-minister, minister of health, mayor), political institutions (European Union), scientific organizations, newspapers, public TV, private TV, social media, medical doctors, and friends (response format: yes/partly/no). All three items concerning trust in politicians (prime-minister, minister of health, mayor) and the items concerning trust in political institutions, scientific organizations, newspapers, and public TV were assigned to the factor trust in institutions (α = 0.828). Two further factors concerning trust, were not considered in the further analysis because of insufficient internal consistency (trust in alternative media, α = 0.279; trust in friends and medical doctors, α = 0.418). A single item was used to assess social norms. Respondents were asked whether the majority of the people they cared about (e.g., family, friends) adhered to specific measures (response format: yes/no).

#### Time-dependent factors

**Figure 2:**
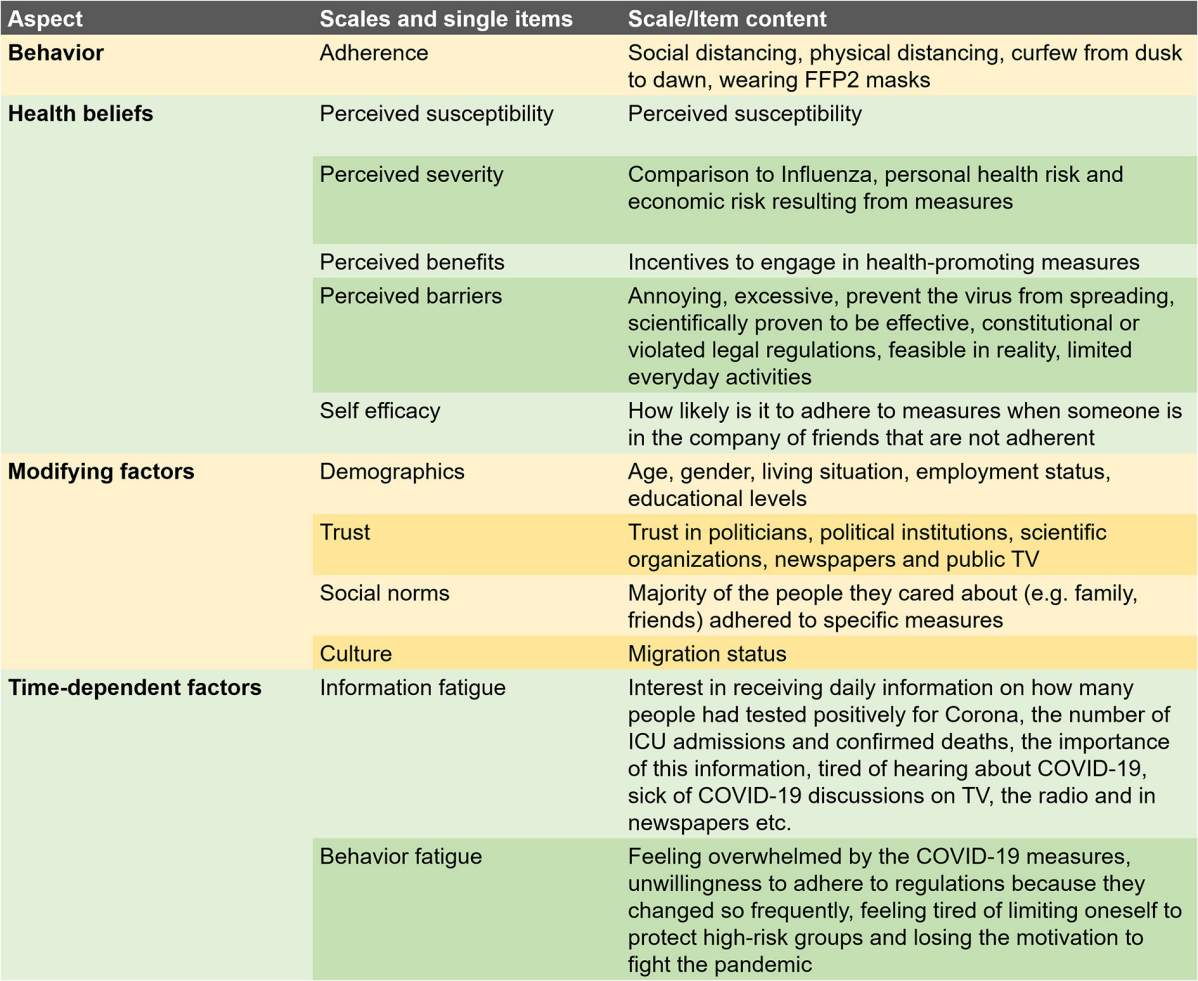
Scales and items used in the survey.

The *corona fatigue* aspect contained all six items from Lilleholt et al.'s (32) corona fatigue questionnaire and has a two-dimensional structure (information fatigue, behavioral fatigue). As the questionnaire was used in a telephone interview, response formats were adapted to take this into account. In this study, the response format was simplified to: agree/partly agree/do not agree. In addition, one item (unwilling to speak to people who downplay the risk of COVID-19) was added and used the same response format. Six further items (response format: yes/partly/no) dealt with fatigue resulting from changing regulations (two items), daily news on the number of people that had tested positively, that had been admitted to an intensive care unit (ICU), or had died (three items), and resignation due to the length of the pandemic (one item). As the factors proposed by Lilleholt et al. (32) only had an internal consistency of α = 0.612 (information fatigue) and α = 0.617 (behavioral fatigue), the 13 items were analyzed together. This resulted in a three-factor model, with two factors showing adequate internal consistency. These two factors were entitled information fatigue and behavioral fatigue (information fatigue: α = 0.766; behavioral fatigue: α = 0.669) (Table 1). The information fatigue factor included items concerning interest in receiving daily information on how many people had tested positively for Corona, the number of ICU admissions and confirmed deaths, as well as the importance of this information. Respondents also rated how tired they were of hearing about COVID-19 and how sick they were of COVID-19 discussions on TV, the radio and in newspapers, etc. Items making up the behavioral fatigue factor were feeling overwhelmed by the COVID-19 measures, unwillingness to adhere to regulations because they changed so frequently, feeling tired of limiting oneself to protect high-risk groups and losing the motivation to fight the pandemic. The third factor was COVID-19 anxiety, which was excluded from further analysis due to insufficient internal consistency (α = 0.374). An overview of the used scales is given in Figure 2.

**Table 1:** Internal consistency of the factors used to evaluate the pandemic.

	**Cronbach's α**
Adherence	0.681
Perceived barriers due to health-promoting measure	0.792
Perceived incentives to engage in health-promoting measures	0.744
Trust in institutions	0.828
Social norms	0.755
Information fatigue	0.766
Behavioral fatigue	0.669

### Survey

The survey was conducted by two professional call centers in two Austrian states from April 20 to June 9. Overall, 500 volunteers that were representative of the population of Carinthia in terms of age, gender, and educational status and were ≥16 years, and 503 from the population of Vorarlberg, participated in the interview study. To achieve this sample size 3,690 persons in Carinthia (response rate 13.6%) and 3,526 in Vorarlberg (response rate 14.3%) were contacted. Participation was voluntary and participants received no incentives.

### Statistics

Baseline characteristics are presented as mean ± SD or median (IQR), as appropriate. Categorical variables are provided as absolute and relative numbers. In a first step univariate linear regression analysis was performed, whereby adherence served as the outcome. Predictors were the factors and the single-item aspects described above, along with sociodemographic variables (age, gender, employment status, living with children, living status, education). Dummy coding was used for categorical variables with more than two categories. To enhance comparability, all factors and single-item aspects apart from age were transformed to range from 0 to 1. To ensure the resulting betas were comparable, the age variable was therefore divided by 100. Univariate significant predictors were checked for multicollinearity (variance inflation factor < 2.5). Remaining variables were included in a multivariate regression analysis (backwards selection). Exploratory data analysis was used to assess the influence of the predictors on the single measures by using logistic regression analysis. For this analysis, univariate significant predictors were also checked for multicollinearity (variance inflation factor < 2.5). The remaining variables were subjected to multivariate logistic regression analysis (backwards selection). SPSS 26 was used for data analysis (36), a value of *p* < 0.05 was considered significant.

## Results

### Demographics

The median age of participants was 50 (38–64) years and 52% of respondents were female. Female respondents were older (female: median 54 years IQR: 41–66; male: 44, 35–62). About 1/3 had a university or high school diploma (EL4 and EL5), while 40% were employed and 30% had retired (Table 2).

**Table 2:** Demographics of participants (*N* = 1,003).

		**Median (IQR)**
		***n* (%)**
Age in years		50 (38–64)
Gender	Female	522 (52.0%)
	Male	479 (47.8%)
	Other	2 (0.2%)
Educational level	EL1: Compulsory education—including school leavers with no certificate of education	106 (10.6%)
	EL2: Apprenticeship	352 (35.3%)
	EL3: College for higher vocational education	214 (21.5%)
	EL4: Academic secondary school	183 (18.4%)
	EL5: University	141 (14.2%)
Employment status	Retired	300 (29.9%)
	Unemployed	28 (2.8%)
	Self-employed	123 (12.3%)
	Employed	398 (39.7%)
	Short-time work	70 (7.0%)
	Homemaker	21 (2.1%)
	Parental leave/sabbatical/care leave	16 (1.6%)
	Student (school, university, etc.)	40 (4.0%)
Living alone	Yes	166 (16.6%)
Living with children	Yes	332 (33.1%)
Migration background	Yes	140 (14.0%)

EL, educational level.

### Health belief model: Descriptive analysis

Overall, respondents' median adherence was 0.75 (IQR: 0.5–1.0). Social norms (median: 1, IQR: 0.67–1.00) and trust in institutions (median: 0.64, IQR: 0.5–0.83) were also rated highly. Respondents rated a COVID-19 infection as more dangerous than an influenza infection (median: 1.0, 0.5–1.0). We also measured self-efficacy (median: 0.5, IQR: 0.5–1.0), personal health risk (median: 0.50, IQR: 0.25–0.75), perceived barriers due to health-promoting measure (median: 0.50, IQR: 0.25–0.75), perceived incentives to engaging in health-promoting measures (median: 0.50, IQR: 0.25–0.75), economic risk stemming from the measures to combat the coronavirus (median: 0.50 IQR: 0.2–0.7), and perceived susceptibility (median: 0.5, IQR: 0.0–1.0). Low ratings were observed for information fatigue (median: 0.4, IQR: 0.2–0.7) and behavioral fatigue (median: 0.25, IQR: 0.00–0.38).

### Influence on adherence

**Figure 3:**
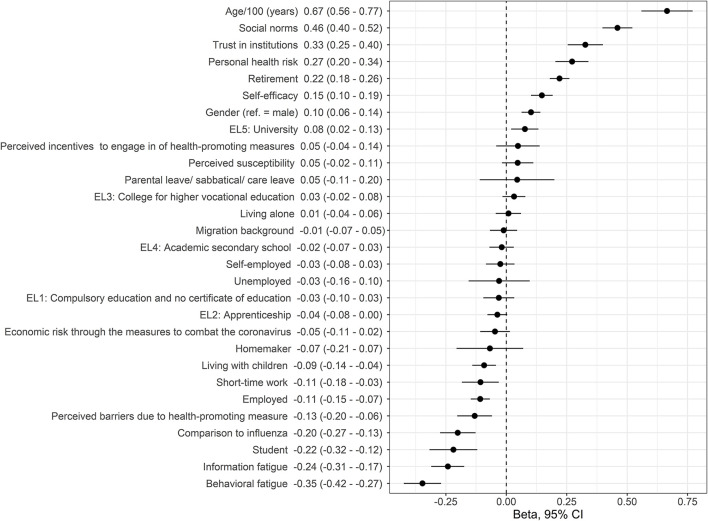
Association between adherence and aspects of the health belief model, modifying aspects, and health beliefs, as derived from univariate regression analysis. Beta-coefficients with 95% confidence intervals are shown. Variables are ordered according to beta-coefficient (EL, educational level).

In a first step, the following variables were significant univariate predictors of adherence to health-promoting behaviors: age, gender, employment status (retirement, employed, short-time work, student), university degree (EL 5), living with children, two perceived severity items (comparison to influenza, personal health risk), self-efficacy, perceived barriers due to health-promoting measure, trust in institutions, social norms, information fatigue, and behavior (Figure 3, Supplementary Table 1).

**Figure 4:**
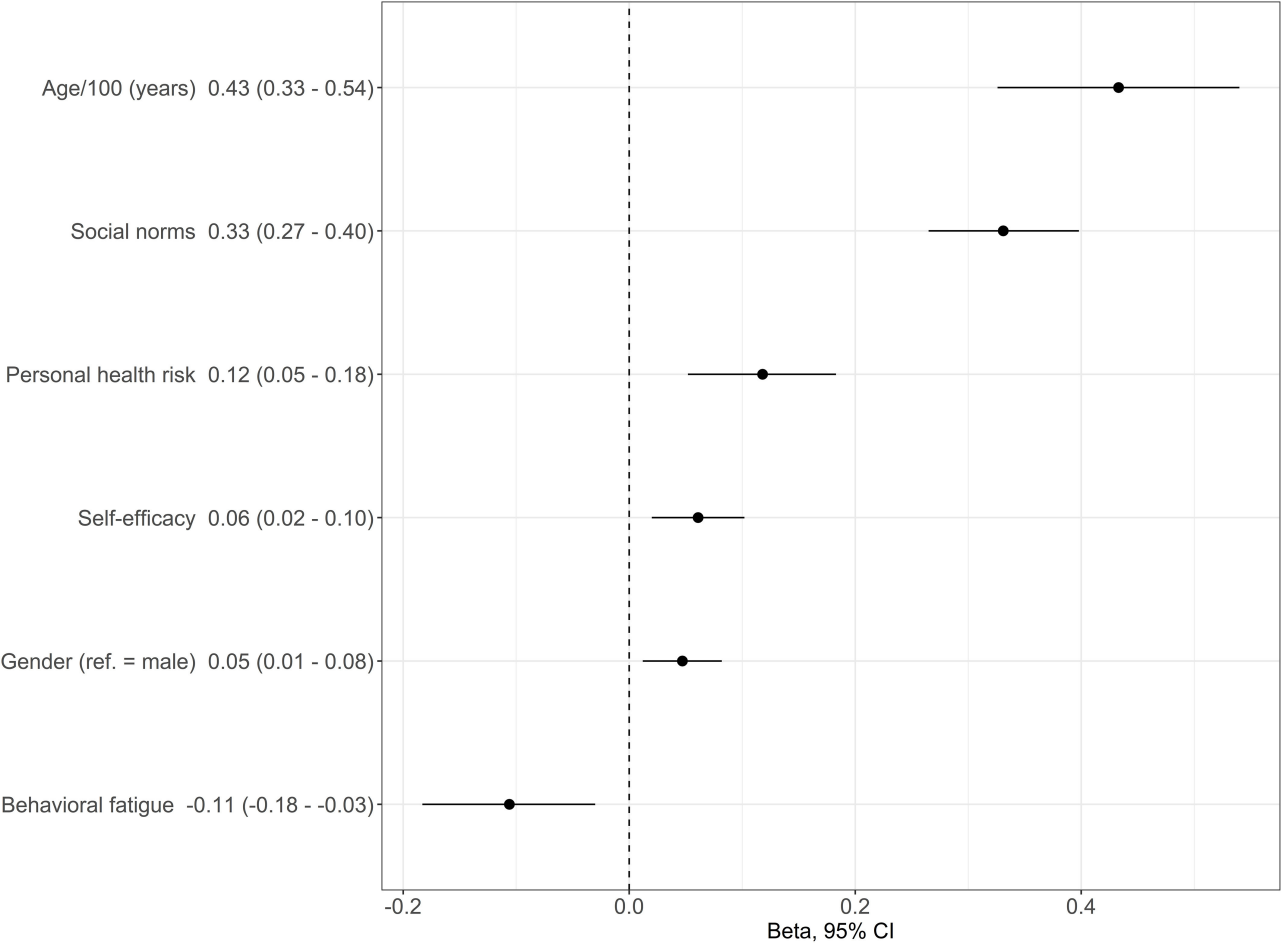
Association between adherence and aspects of the health belief model, modifying aspects and health beliefs, as derived from multivariate regression analysis. Beta-coefficients with 95% confidence intervals are shown.

In a second step, multivariate regression analysis indicated that six independent predictors explained 29% of the variance in adherence [$R_{\text{adjusted}}^{2}$ = 0.285, *F*_(1)_ = 59.85, *p* < 0.001]. Higher age (β: 0.43 95%CI: 0.33–0.54; *p* < 0.001), social norms (β: 0.33 95%CI: 0.27–0.40; *p* < 0.001), perceived personal health risk (β: 0.12 95%CI: 0.05–0.18; *p* < 0.001), self-efficacy (β: 0.06 95%CI: 0.02–0.10; *p* = 0.002), female gender (β: 0.05 95%CI: 0.01–0.08; *p* = 0.002), and decreased behavioral fatigue (β: −0.11 95%CI: −0.18 to −0.03; *p* = 0.045) were associated with increased health-promoting behavior (Figure 4).

### Influence on single measures of adherence

Multivariate regression analysis of individual measures indicated that two to seven independent predictors explained 9–27% of variance. Twelve different predictors were included in the final six models. No predictor was included in all final models. The predictors that were most often included were behavioral fatigue (four times) and age (three times) (Table 3, Supplementary Figure 2; univariate results: Supplementary Figure 1).

**Table 3:** Association between individual measures and aspects of the health belief model, modifying aspects and health beliefs, as derived from multivariate regression analysis.

	**Physical distancing**	**Wearing FFP2 masks**	**Respecting dusk-to-dawn curfew**	**Social distancing**	**Testing when symptoms are present**	**Testing**
Age	20.5 (6.0–71.3)		17.9 (1.7–192.5)	5.3 (1.2–22.9)		
Female					2.3 (1.0–5.5)	
Employed				0.6 (0.4–0.9)		2.1 (1.4–3.3)
Living with children		0.4 (0.2–0.9)				
Perceived health risk	2.6 (1.3–5.2)			2.7 (1.2–6.0)		
Comparison to influenza				4.6 (1.8–11.9)		0.3 (0.1–0.5)
Perceived incentives to engage in health-promoting measures			3.5 (1.4–9.3)			
Information fatigue			4.6 (1.4–14.8)			0.2 (0.1–0.4)
Behavioral fatigue		0.2 (0.1–0.8)		0.3 (0.1–0.6)	0.1 (0.0–0.3)	0.4 (0.2–1.0)
Social norms	5.2 (2.7–10.1)			8.6 (4.2–17.9)		
Self-efficacy			2.3 (1.1–4.8)	2.2 (1.4–3.6)		
Trust in institutions		10.0 (2.8–35.6)				

OR with 95% confidence intervals are shown.

## Discussion

**Figure 5:**
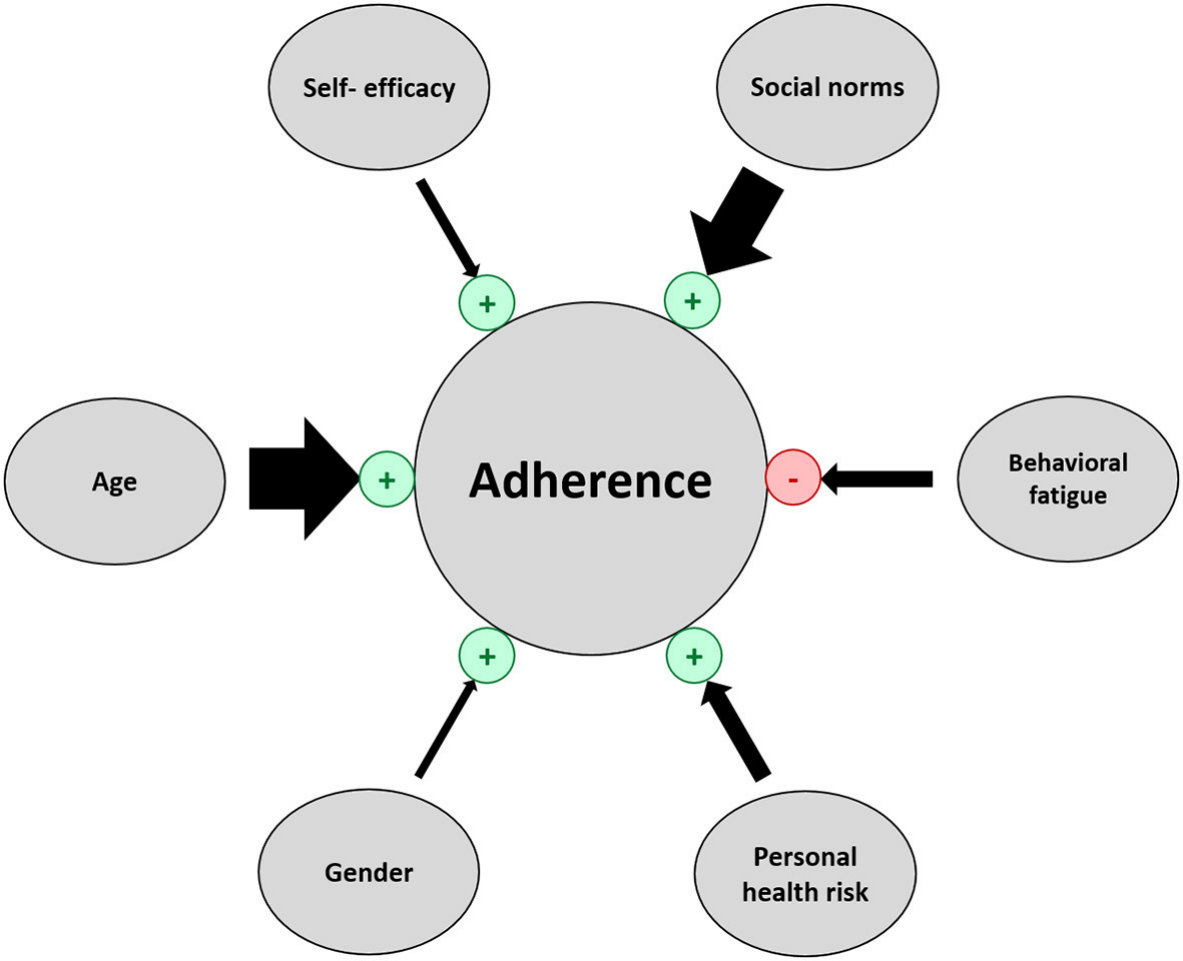
Predictors of adherent behavior. Arrow width corresponds to the absolute value of beta (regression coefficient). Predictors that increase adherence are marked in green “+” and predictors that decrease adherence are marked in red “–”.

In this representative cross-sectional telephone survey conducted in Austria, increasing age, social norms, perceived personal health risk, self-efficacy, female gender, and lower behavioral fatigue were independent predictors of greater adherence to a bundle of measures such as social distancing, physical distancing, respecting dusk-to-dawn curfews and wearing FFP2-masks (Figure 5). The predictors differed depending on the measure.

### Age and gender

The survey revealed that higher age and female gender were independent predictors of adherent behavior. Even though previously published studies were inconclusive, a large percentage of studies support our results (23, 24, 37–39). For example, one Canadian study of over 2,000 persons between 18 and 100 years old showed that age and male gender were associated with lower adherence to different COVID-19 protective measures such as working remotely from home, social distancing, and maintaining a physical distance of 2 m from others (23). Another study that used cluster analysis to compare adopters and non-adopters of COVID-19 measures in 5,893 persons between 18 and 94 years old confirmed that older and female persons had lower odds of being in the non-adapter cluster (37). No influence of age or gender was found in a survey of elderly persons (aged over 60 years), which may reflect homogeneity across these variables within the study group (22). Wolfe's paper, which focused on age differences in COVID-19 risk-taking, also revealed that risk perception for the self and others partially mediated the effect of age differences on taking risks (38). One reason why the younger population seems to be less adherent to protection measures may be that they are less vulnerable to the consequences of an infection with SARS-Covid-2. It is well-known that the likelihood of complications, hospitalization, and death is dependent on age, and this has been extensively communicated in the media and by public institutions. Another reason may be that people of younger age are still actively involved in the workforce and may frequently feel that the risk of financial loss offsets concerns about becoming infected.

Results on the effect of gender differences are contradictory. One study of 21,649 persons from eight OECD countries (Australia, Austria, France, Germany, Italy, New Zealand, the UK, and the US) underpins our findings that women have been more adherent to pandemic rules in all countries and take the pandemic more seriously (40). In addition, the paper by Abd Elhameed Ali et al., which also presented results from over 700 people, shows female gender to be positively related to better knowledge about COVID-19 measures and greater adherence to containment measures (26). In contrast, an online survey of 893 Brazilians by Carvalho and Machado that was primarily concerned with the correlation of adherence to pandemic rules and psychopathy traits showed no gender differences (25). In summary, it can be seen that the influence of gender and age found in our study is found in many but not all studies.

### Social norms

In our study we could also show that social norms are strongly associated with increased health-promoting behavior. Even though the results found in the literature appear to be inconsistent (20, 28), this paper supports recent findings indicating that social norms have a significant impact on adherence to COVID-19 measures in the general population (31, 41). In the Corona pandemic, social norms have played an important role in reducing individual transmission risk, as well as the transmission rate in the population as a whole. As studies suggest that social norms and social identities influence behavioral changes, it is important to mention the potential impact of influencing social norms and attitudes to specific COVID-19 measures, especially in vulnerable groups. According to Neville et al. (42), public health messages aimed at changing behaviors should focus on specific groups and present the desired behavior, without including any reference to unwanted behaviors. These messages should be presented by people that are perceived as “one of us.” The intended behavioral change should be framed in an identity-affirming manner, and group members should be seen to change their behavior without losing their influence and without polarization within the group. As social norms are formed by all members of a group, each individual has an influence. According to a review by Tankard and Paluck (43), understanding norms requires information on individual behavior, the group as a whole, and institutional signals. Each of these may be influenced by COVID-19 measures and information strategies that focus on providing consistent information that takes into account group identities and aim to enhance people's self-efficacy. Even if an influence of social norms on behavior cannot be found in all studies, as in our study, social norms may play an important role in adherence.

### Perceived health risk

Another factor we found to have an impact on adherence is perceived personal health risk. This means the more dangerous the virus is considered to be, the more willing a person is to take protective action. These findings confirm further cross-sectional studies such as Lang et al. (37) showing that people who were unconcerned that an infection with the virus might lead to severe symptoms had higher odds of being non-adopters of non-pharmaceutical preventive interventions. Furthermore, in a sample of over 6,600 persons in the US, Bruine de Bruin and Bennett also found people that considered high risk to be associated with an infection with the coronavirus to be more likely to adopt protective behaviors (44). It is interesting, however, that the perceived threat of a SARS-COVID-19 infection having serious repercussions seems to have declined over the course of the pandemic. Results from a longitudinal survey from three rounds of interviews in Spain conducted between July and November 2020 revealed that the perceived threat of becoming seriously ill if infected with COVID-19 infected decreased over time, although the probability of becoming infected remained stable (7). In addition, another study involving 30,000 interviews conducted in 39 rounds in Hong Kong also analyzed temporal changes in the perceived severity of the disease and found it to be positively associated with the incidence of infected people (33).

Besides the perceived health risk, there are also known differences in the health risk due to COVID-19 between groups. In the case of diabetics, for example, it is possible to determine the individual risk with the help of models (45). Furthermore, modern technologies can be useful in early diagnosis and accurate classification of COVID-19 patients (46) and combat COVID-19 (47, 48).

### Behavioral fatigue

In our study, lower behavioral fatigue was associated with greater health-promoting behavior. These results have been confirmed in further studies showing that behavioral fatigue associated with the Corona pandemic impacted people's adherence to measures to reduce transmission risk in the population (5, 32, 33).

Martinez-Garcia et al. analyzed data from a survey of 20,054 persons that was conducted in Spain from April to September 2020 and showed that adherence to containment measures declined over time (49). While they found that the psychological impact was the most important predictor of adherence to containment measures in the beginning, the economic impact played a greater role at the end of the period under review. The authors recommended the use of psychological and economic support programs to enhance adherence in the population. Reicher and Drury also concluded that lower adherence may be related to the availability of financial resources in the population and not only to psychology. Measures to counteract behavioral fatigue should therefore consider the specific needs of communities (50).

Liao et al. showed that psychological fatigue is also associated with public confidence in government, and psychological distress. Thus, fatigue is not only a predictor of adherence but also has an effect on other health-related aspects and may be influenced by official measures and strategies (33).

Based on qualitative data from our survey (not shown), we would also suggest that behavioral fatigue is influenced by changes in behavior. People may, for example, develop strategies to reduce their individual transmission risk (e.g., daily testing rather than wearing FFP2 masks), which may explain why some people do not follow all governmental measures. Behavioral fatigue may therefore be lower and adherence higher than shown in the results, as it is generally based on a measurement of adherence to concrete measures.

### Self-efficacy

Comparable to other studies [e.g., (51)] self-efficacy was also found to be a predictor of adherence to COVID-19 measures. Even though the other predictors in the model have a larger influence on adherence, self-efficacy nonetheless plays an important role in dampening the Corona pandemic, as self-efficacy enhances adherence and reduces an individual's risk of infection. Moreover, COVID-19-related self-efficacy is also reported to be positively correlated with mental health, preventive behavior, and knowledge about COVID-19 (52). Additionally, enhancing self-efficacy not only influences an individual's transmission risk, but may also reduce the rate of new infections in the population as a whole. This is because self-efficacy appears to strengthen social norms and lead to more preventive behavior. On the other hand, Alemany-Arrebola et al. found that self-efficacy was sometimes negatively affected by aspects related to COVID-19, such as perceived stress associated with the pandemic, confinement, and critical events (illness and death of a relative/friend due to COVID-19) (53). These aspects increase individuals' anxiety levels and reduce their self-reported perceptions of (academic) self-efficacy. In summary, self-efficacy is an important aspect of adherence that was also found in other studies.

## Strengths and limitations of the study

The study has several strengths and limitations. The cross-sectional telephone study was performed by trained and experienced interviewers. Participants were representative of the broader population above 16 years of age in terms of age, gender, and educational status. As only about 14% of contacted persons were willing to participate in a telephone interview, a self-selection bias cannot be ruled out. This bias—also called the volunteer effect—is characterized by differences in the likelihood that certain people will answer a survey, depending on e.g., the content or design of the survey, offered incentives, their personality, socio-economic status, and gender (54–56). In our sample, 38.9% of respondents said they are tired of hearing about COVID-19. It cannot be ruled out that the overall number of people that are tired of hearing about COVID-19 is higher and that these people are less likely than others to answer a survey on COVID-19. Nevertheless, since the aim of this study was not to analyze the percentage of people that are adherent but to analyze the underlying factors that influence adherence, this self-selection bias should not have affected results. It is also possible that some of the questions were answered differently than they would have been in paper-pencil or online surveys. It has been shown [e.g., (57, 58)] that the method of survey influences responses in different ways, but with no specific bias in favor of a specific method. Since we wanted to reach older people and face-to-face interviews were not possible due to the pandemic, we decided not to use online surveys, so that people with no internet account, who tend to be older, could also be reached.

One shortcoming of our study is that the survey was performed in spring 2021 and at a time when the infection rate was low and the population expected protective measures to be relaxed during the upcoming summer season. Nevertheless, we have assumed that while the amount of corona fatigue may change over time, its impact in terms of β or OR will be comparable over time. This is supported by Lang et al. (37) who clustered data from almost 4,500 persons from a Canadian cross-sectional survey and found similar rates among adopters and non-adopters of COVID-19 measures. He effectively confirmed our results as non-adopters tend to be younger males that are less worried about COVID-19.

## Conclusion

The results of this representative Austrian cross-sectional telephone study show that when the health belief model is combined with aspects that vary over time and other modifying aspects, it can make a valuable contribution toward explaining adherence (Table 4). Age, social norms, perceived personal health risk, self-efficacy, female gender, and lower behavioral fatigue increase overall adherence to government measures to control the COVID-19 pandemic. Furthermore, adherence to individual measures was also influenced by other aspects of the model (e.g., wearing FFP2-masks by trust in institutions, and dusk-to-dawn curfews by information fatigue), showing that strategies need to be tailored depending on what particular behavior is being targeted.

**Table 4:** Summary table.

What previous studies found:	• The health belief model is widely used to develop a conceptual understanding of individual adherence to preventive activities. • Inconsistent results have been found for socio-demographic variables such as age, gender, education, and social norms. • Another aspect that has frequently been examined in connection with the pandemic is corona fatigue.
What this study adds:	• In this study, the findings from the health belief model are examined together with findings from other areas. • It follows that both aspects of health belief model (e.g., social norms) and other aspects (e.g., corona fatigue) are important for adherence.

Strategies to improve adherence should therefore be adapted depending on the goal (overall adherence or adherence to individual measures) and on the group of persons that is being targeted (e.g., informal and formal group leaders or vulnerable groups) rather than being addressed to everyone. Furthermore, institutional signals play an important role and, if used imprudently, can thwart efforts to change behavior.

## Data availability statement

The raw data supporting the conclusions of this article will be made available by the authors, without undue reservation.

## Ethics statement

The studies involving human participants were reviewed and approved by Ethics Committee of the state of Carinthia/Austria (M2021-15). Written informed consent from the participants' legal guardian/next of kin was not required to participate in this study in accordance with the national legislation and the institutional requirements.

## Author contributions

AS, AA, KJ, and DS developed a concept for the paper. AA conducted the analysis. AS, AA, CK, DS, and PE were responsible for the writing process. KJ and CK contributed to the analysis. AS, AA, CK, KJ, and PE contributed to interpreting results and drafting the manuscript. All authors critically reviewed all drafts of the manuscripts and approved the final manuscript before submission.

## Funding

The study was funded by the Austrian Federal Ministry for Social Affairs, Health, Care and Consumer Protection.

## Conflict of interest

Author DS was employed by Austrian Agency for Health and Food Safety Ltd. AGES. The remaining authors declare that the research was conducted in the absence of any commercial or financial relationships that could be construed as a potential conflict of interest.

## Publisher's note

All claims expressed in this article are solely those of the authors and do not necessarily represent those of their affiliated organizations, or those of the publisher, the editors and the reviewers. Any product that may be evaluated in this article, or claim that may be made by its manufacturer, is not guaranteed or endorsed by the publisher.
