# Supplementary material for: Predictors for adherent behavior in the COVID-19 pandemic: A cross-sectional telephone survey
Source: Front Public Health. 2022 Oct 20;10:894128. doi: 10.3389/fpubh.2022.894128 (PMC9632415; doi:10.3389/fpubh.2022.894128)
Supplement: Supplementary file 1 [file Data_Sheet_1.docx]

**Predictors for adherent behavior in the COVID-19 pandemic: A cross-sectional telephone survey**

Supplemental Material

**Supplemental Table 1** Association between adherence and aspects of the health belief model, modifying aspects and health beliefs, as derived from univariate regression analysis. Beta-coefficients with 95% confidence intervals are shown.

|  | p-value | β (95%CI) |
| --- | --- | --- |
| Age/100 | <.001 | .665 (.559 - .771) |
| Gender (Ref: male) | <.001 | .102 (.063 - .141) |
| Educational Level (EL) |  |  |
| EL1: Compulsory education Including school leavers with no certificate of education | .343 | -.031 (-.095 - .033) |
| EL2: Apprenticeship | .075 | -.037 ( -.078 - .004) |
| EL3: College for higher vocational education | .191 | .032 (-.016 - .079) |
| EL4: Academic secondary school | .453 | -.019 (-.070 - .031) |
| EL5: university | .008 | .077 (.020 - .133) |
| Employment status |  |  |
| Retirement | <.001 | .220 (.180 - .261) |
| Unemployed | .645 | -.030 (-.156 - .097) |
| Self-employed | .410 | -.025 (-.084 - .034) |
| Employed | <.001 | -.108 (-.147 - -.068) |
| Short-time work | .006 | -.107 (-.184 - -.031) |
| Homemaker | .336 | -.068 (-.206 - .070) |
| Parental leave/ sabbatical/ care leave | .570 | .045 (-.110 - .199) |
| Student | <.001 | -.219 (-.318 - -.120) |
| Living alone | .748 | .009 (-.044 - .061) |
| Living with children | .001 | -.066 (-.104 - -.029) |
| Migration background | .693 | -.011 (-.068 - .045) |
| Perceived barriers due to health-promoting measures | <.001 | -.131 (-.203 - -.059) |
| Perceived incentives to engage in of health-promoting measures | .293 | .048 (-.042 - .138) |
| Trust in institutions | <.001 | .327 (.254 - .400) |
| Social norms | <.001 | .460 (.398 - .522) |
| Information fatigue | <.001 | -.242 (-.311 - -.174) |
| Behavioral fatigue | <.001 | -.347 (-.424 - -.270) |
| Comparison to influenza | <.001 | .201 (.127 - .274) |
| Personal health risk | <.001 | .272 (.203 - .340) |
| Economic risk due to measures to combat the coronavirus | .136 | -.047 (-.108 - .015) |
| Self-efficacy | <.001 | .147 (.102 - .192) |
| Perceived susceptibility | .160 | .047 (-.018 - .112) |

*EL … educational level*

**Supplemental Table 2.** Association between adherence and aspects of the health belief model, modifying aspects and health beliefs, as derived from multivariate regression analysis. Beta-coefficients with 95% confidence intervals are shown.

|  | p-value | β (95% CI) |
| --- | --- | --- |
| Age/100 | <.001 | .420 (.310 - .529) |
| gender (ref. = male) | .002 | .047 (.012 - .083) |
| Personal health risk | <.001 | .030 (.013-.047) |
| Self-efficacy | .002 | .033 (.012 - .054) |
| Social norms | <.000 | .350 (.281 - .419) |
| Behavioral fatigue | .045 | -.053 (-.092 - -.014) |

*CI: confidence interval; ref: reference*

*
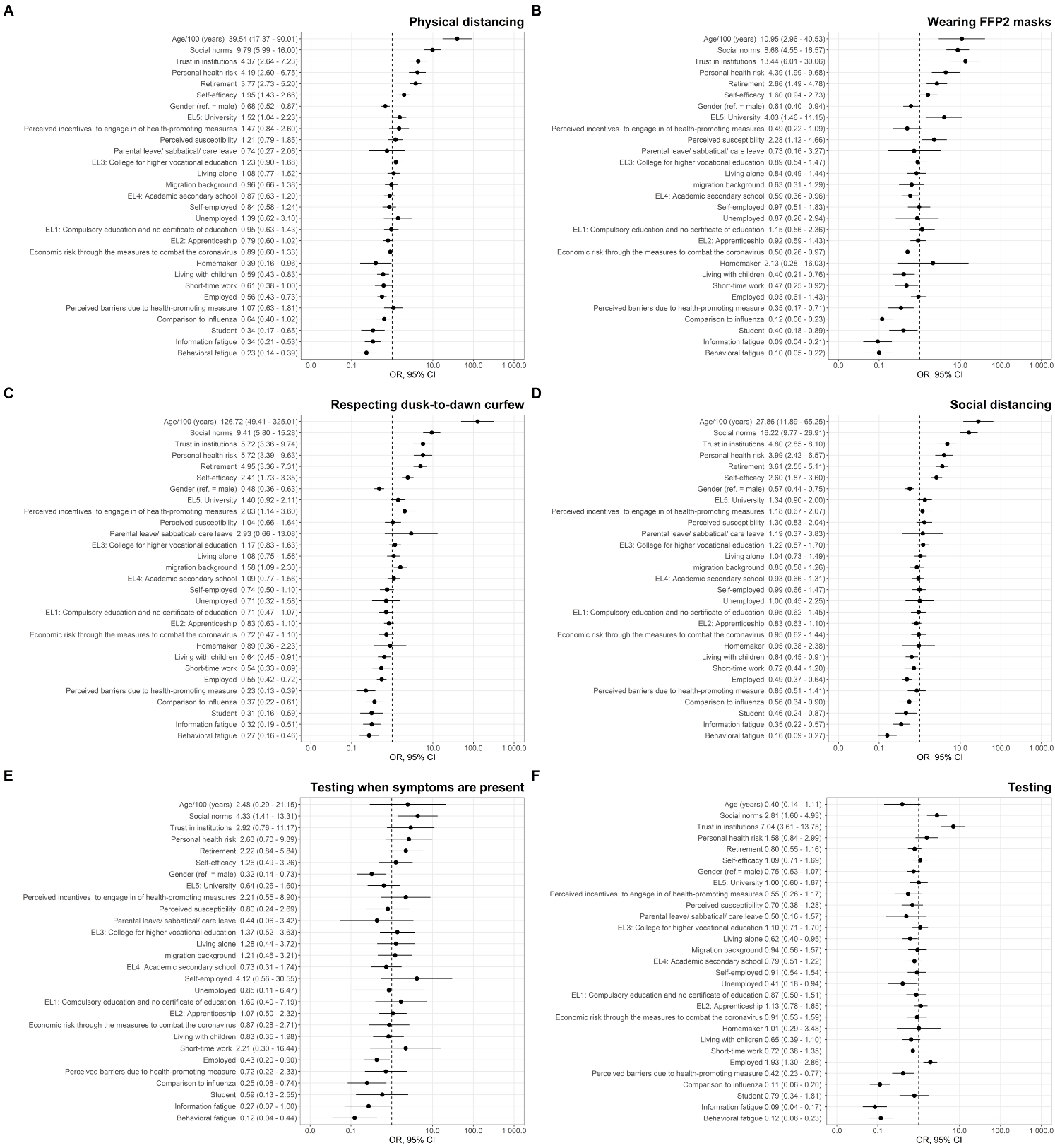
*

**Supplemental FIGURE 1.** Association between adherence to individual measures and aspects of the health belief model, modifying aspects and health beliefs, as derived from univariate regression analysis. Odds Ratios with 95% confidence intervals are shown (EL: educational level; OR: odds ratio). Order of the variables is the same as in figure 2.

**
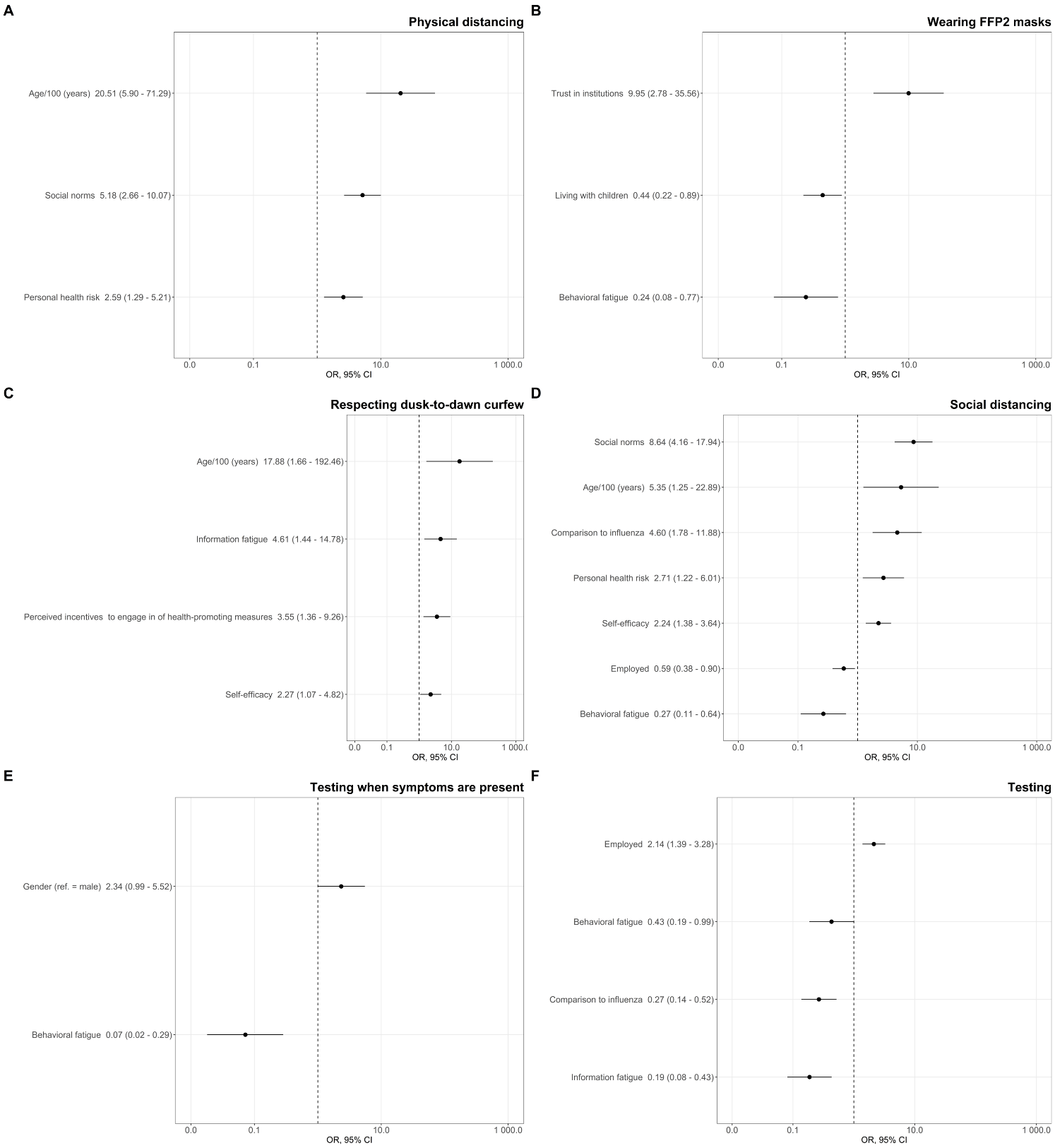
**

**Supplemental Figure 2.** Multivariate association between adherence to individual measures and aspects of the health belief model, modifying aspects and health beliefs. Odds Ratio with 95% confidence intervals are shown (OR: Odds Ratio).

**Supplement – Questionnaire**

This questionnaire was used in German. This version was translated from German to English for a better understanding of the content of the manuscript. No backward translation was carried out. Before using this questionnaire in English a backward translation and a pilot test should be performed.

**
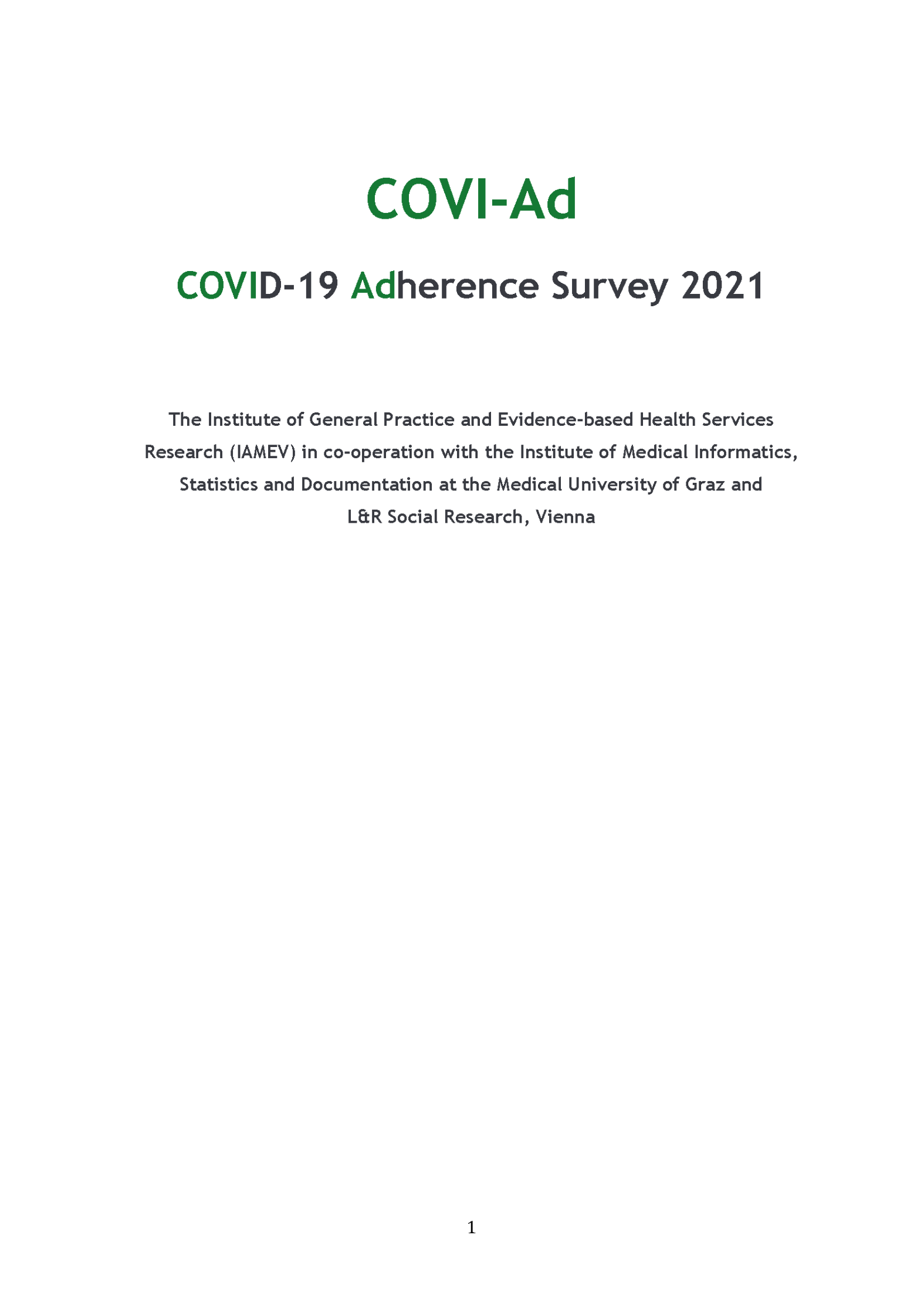
**

**
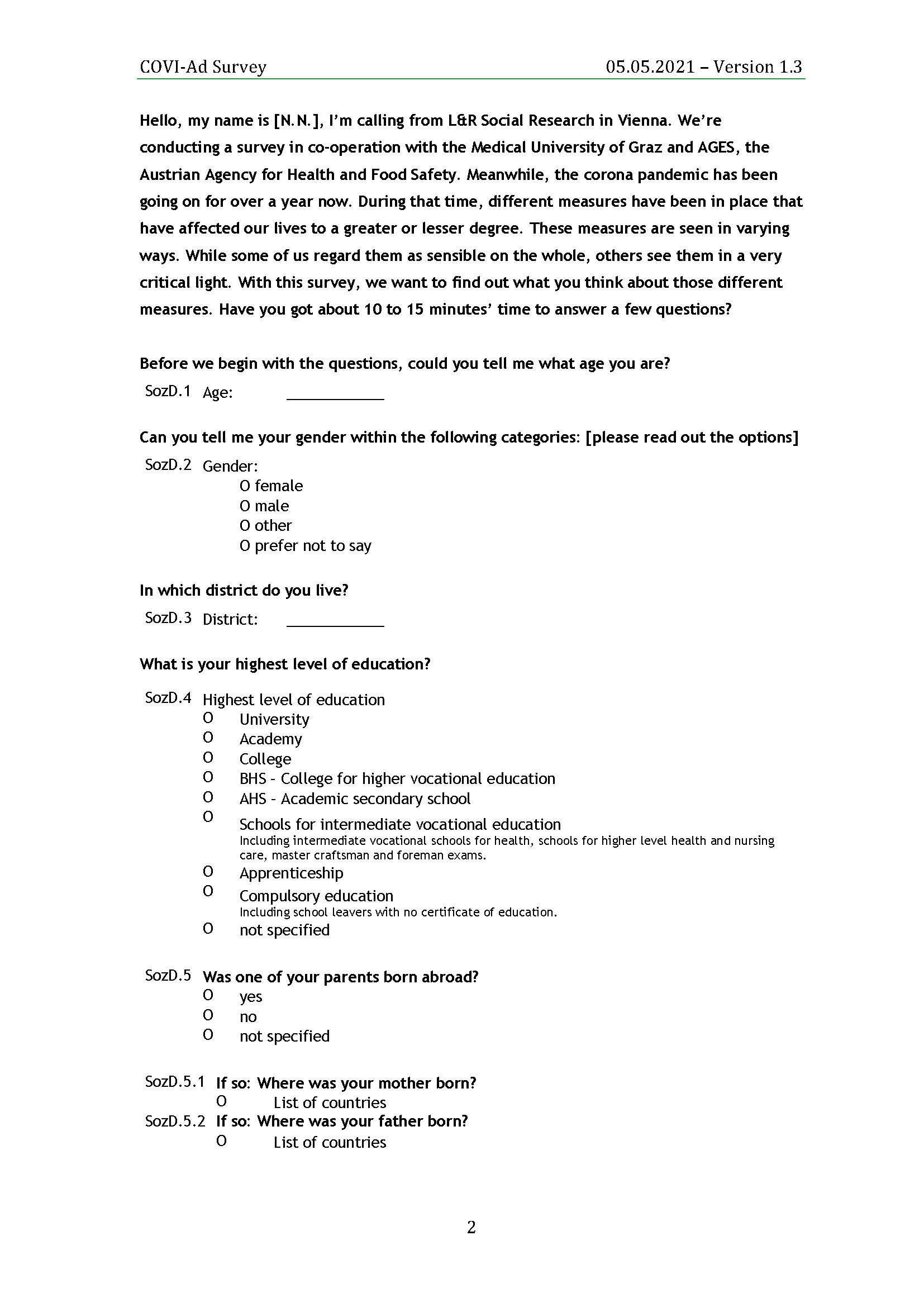

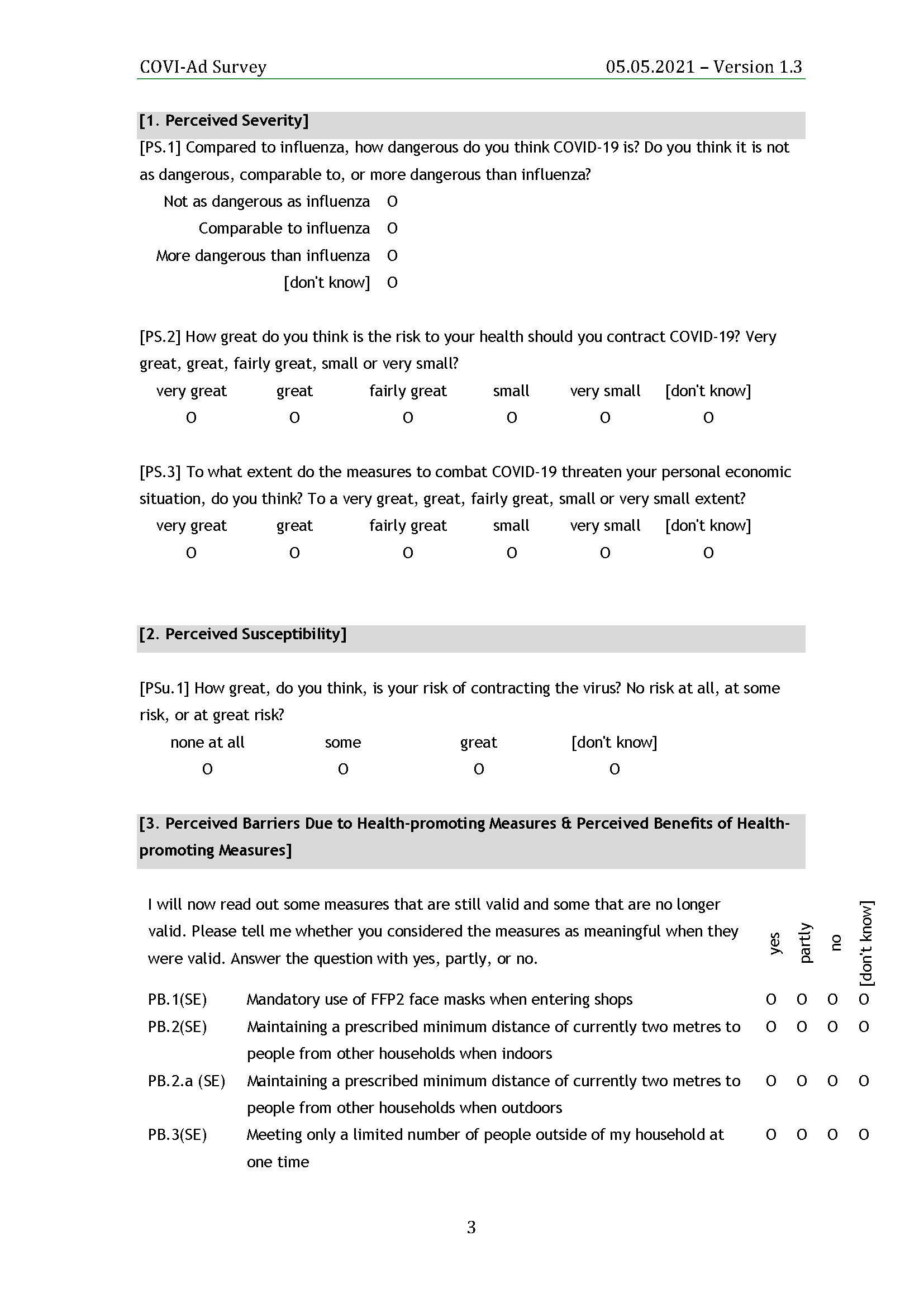
**



**
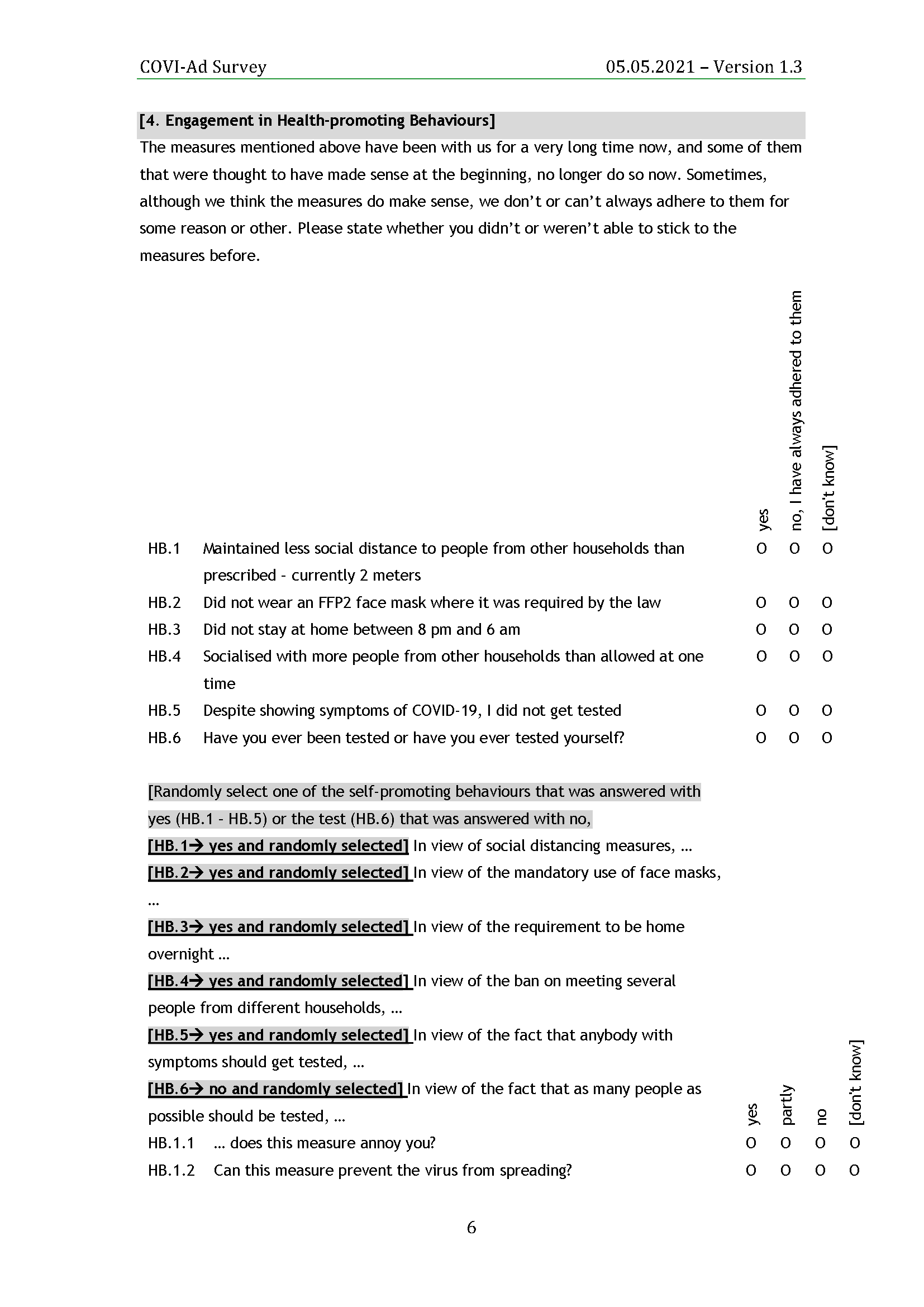
**

**
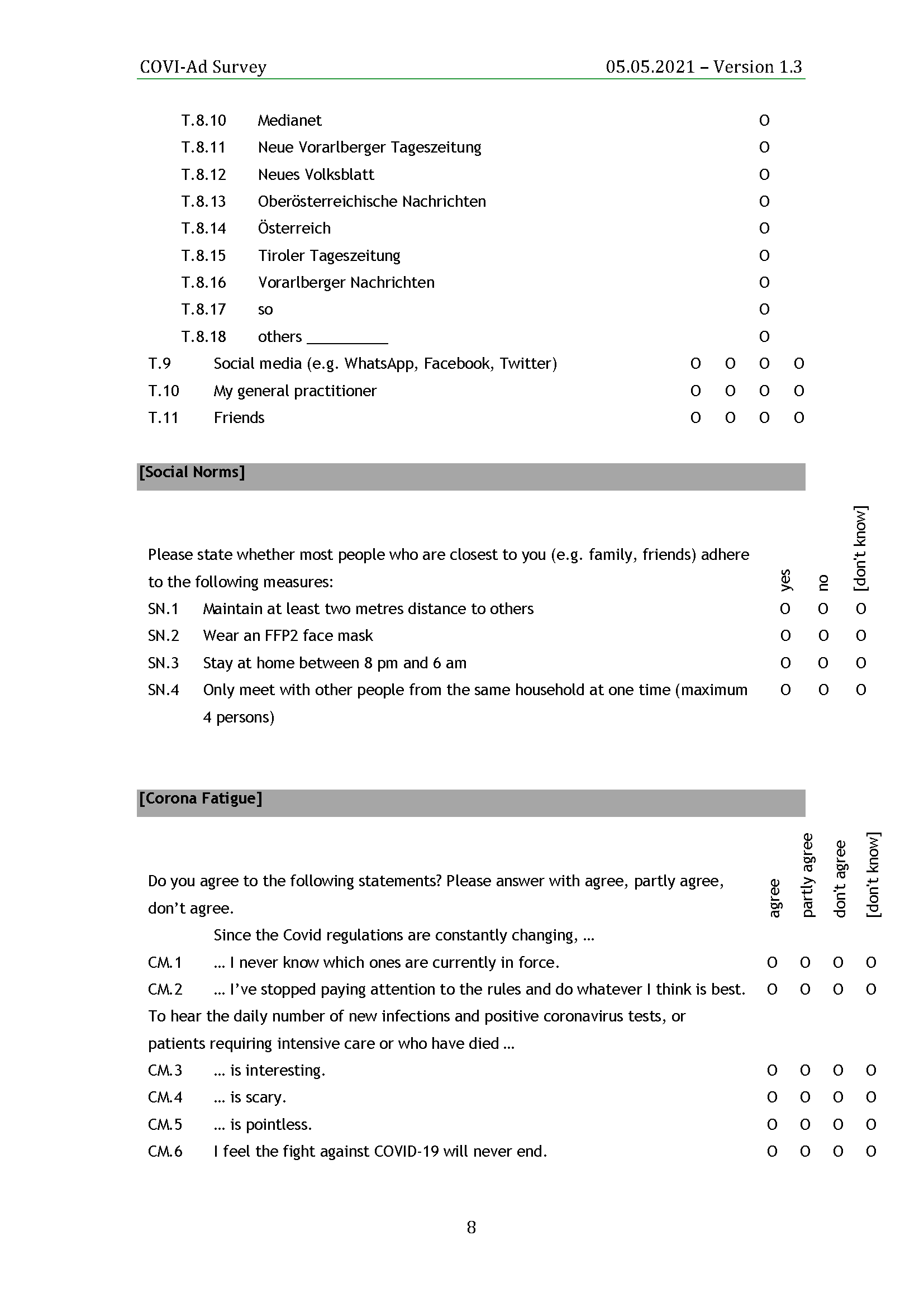

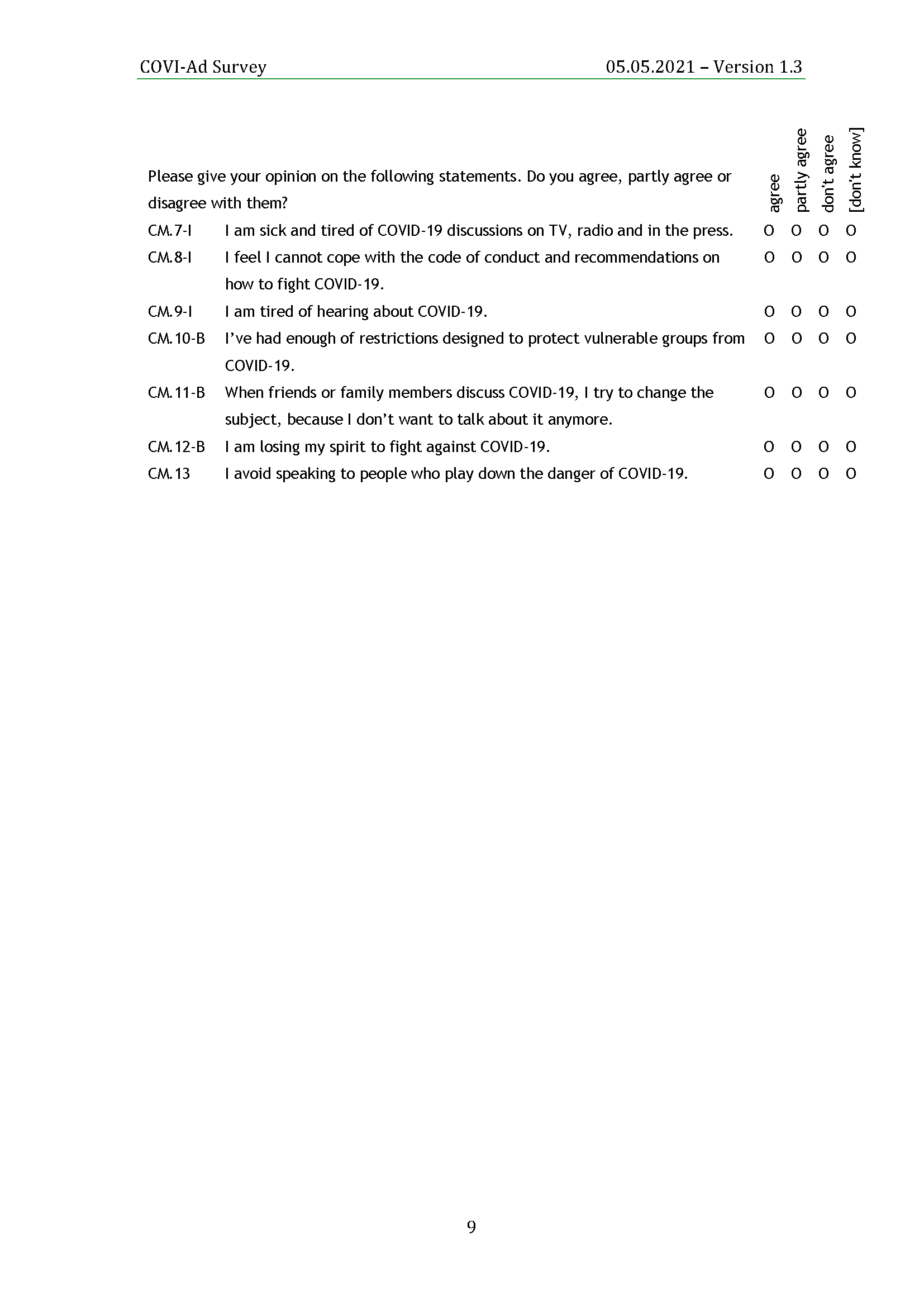
**
